# A New Era in Obesity Treatment: The Evolution of Antiobesity Medications (AOMs) Based on Clinical Trials

**DOI:** 10.1111/1753-0407.70115

**Published:** 2025-06-17

**Authors:** Danlu Liu, Yi Chen

**Affiliations:** ^1^ Division of Gastrointestinal Surgery, Department of General Surgery West China Hospital, Sichuan University Chengdu Sichuan China

**Keywords:** anti‐obesity medications (AOMs), diabetes, obesity

**Abbreviations**
AOMs: antiobesity medications
GDF15: growth differentiation factor 15
GLP‐1: glucagon‐like peptide‐1
NuSH: nutrient‐stimulated hormone

To the Editor,

Obesity represents a critical global health challenge, marked by escalating prevalence and comorbidities such as cardiovascular disease, type 2 diabetes, chronic kidney disease, musculoskeletal disorders, and certain cancers [1]. The continuous development of antiobesity medications (AOMs), which demonstrate significant weight loss effects, presents new opportunities for the treatment of obesity [2]. On August 8, 2024, a search of the Informa Database identified a total of 2120 trials for AOMs, indicating that the research on AOMs is active and receiving significant attention.

Over the past 30 years, the number of clinical trials on AOMs has experienced significant fluctuations, peaking in 2023–2024 (Figure 1A). The number of clinical trials entering phases III–IV and phases I–II/III was comparable (48.5% vs. 50.9%), underscoring the development of novel AOMs derived from established therapies. Eventually, a total of 20 indications were identified in these trials, of which the top 10 were listed in Figure 1B. The majority of indications were Non‐diabetic overweight or obesity (41.6%), followed closely by Diabetes‐related overweight or obesity (41.0%). Among trials targeting non‐diabetic overweight or obesity, only 35.6% reached phase III/IV, indicating this field is still in early‐stage exploration. Figure 1C illustrates the distribution of clinical trials for AOMs based on their mechanisms of action and targets. Nutrient‐stimulated hormone (NuSH) single receptor agonists dominate the landscape, accounting for 26.14% of trials, highlighting their significant research focus. Other AOMs, which target leptin, ghrelin, mitochondrial uncouplers, and growth differentiation factor 15 (GDF15), follow closely at 19.94%. In recent years, as the number of clinical trials on AOMs has steadily increased, the proportion of combined therapies has also risen, suggesting that this will be a future trend (Figure 1D). Recent advancements have been characterized by a surge in clinical trials targeting various mechanisms of action, as depicted in Figure 1E. Glucagon‐like peptide‐1 (GLP‐1) receptor agonists have emerged as a leading class, with Semaglutide and Liraglutide being the most extensively studied drugs classified as NuSH single receptor agonists. Meanwhile, peptides and molecular conjugates with dual‐agonist and triple‐agonist actions, such as Tirzepatide and Retatrutide, are currently under investigation and appear to have the strongest potential as anti‐obesity medications [3]. Advances in incretin biology have driven the development of approved GLP‐1 receptor agonists over recent decades. To date, the FDA has approved three NuSH‐based AOMs: Liraglutide, Semaglutide, and Tirzepatide.

**FIGURE 1:**
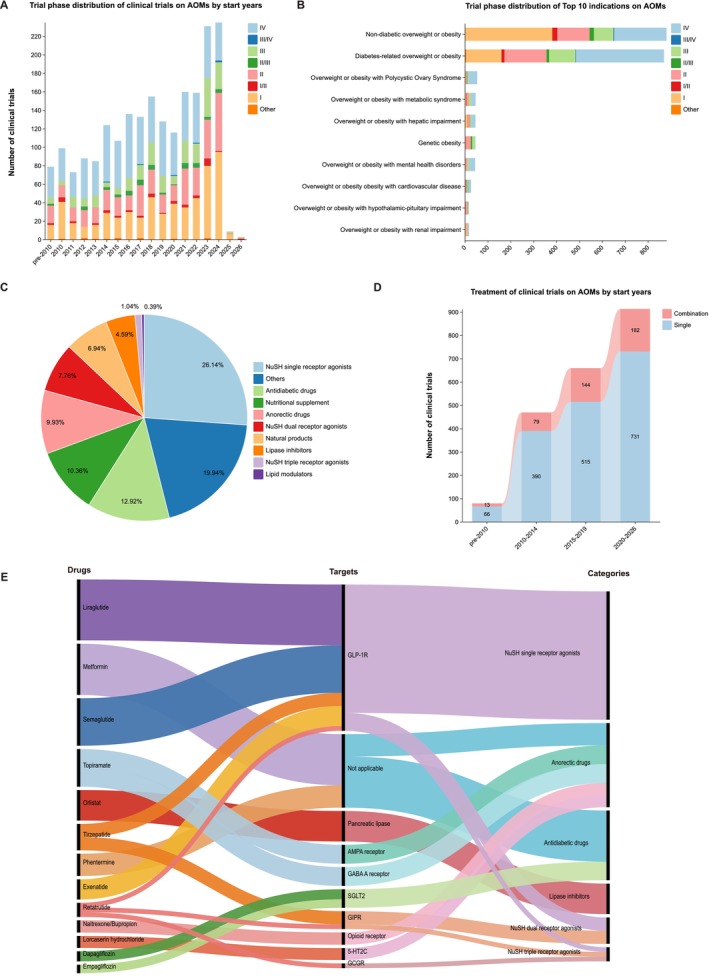
Clinical trial landscape of antiobesity medications (AOMs) worldwide. (A) Trial phase distribution of clinical trials on AOMs by start years; (B) Trial phase distribution of Top 10 indications on AOMs; (C) Classification of AOMs by mechanisms of action and targets; (D) Treatment of clinical trials on AOMs by start years; (E) Top drugs and mechanisms of action. 5‐HT2C, 5‐hydroxytryptamine receptor 2C; AMPA, α‐amino‐3‐hydroxy‐5‐methyl‐4‐isoxazolepropionic acid; GABA A, γ‐aminobutyric acid type A; GCGR, glucagon receptor; GIPR, glucose‐dependent insulinotropic polypeptide receptor; GLP‐1, glucagon‐like peptide‐1; NuSH, nutrient‐stimulated hormone; SGLT2, sodium‐glucose co‐transporter 2.

Additionally, further management of obesity necessitates personalized therapies and multimodal strategies, as long‐term maintenance of weight loss is challenging for most people. Combination therapies, like the integration of AOMs or bariatric surgery, alongside lifestyle modifications, represent the future direction of treatment development. Concurrently, research focusing on non‐diabetic overweight or obese patients has gained traction, reflecting growing public interest in obesity and the ongoing development of novel AOMs.

In conclusion, as novel AOMs are developed and clinical experience grows, obesity treatment will become more effective, helping patients improve their quality of life and alleviating the burden of obesity‐related comorbidities.

## Author Contributions

**Danlu Liu:** conceptualization, data curation, formal analysis, investigation, methodology, writing original draft. **Yi Chen:** supervision, validation, writing – review and editing, project administration, funding acquisition. All authors have made substantial contributions to the conception, design, execution, or interpretation of the reported study, in line with the latest guidelines of the International Committee of Medical Journal Editors (ICMJE).

## Ethics Statement

The authors have nothing to report.

## Consent

The authors have nothing to report.

## Conflicts of Interest

The authors declare no conflicts of interest.

## References

[1] A. Elmaleh‐Sachs , J. L. Schwartz , C. T. Bramante , J. M. Nicklas , K. A. Gudzune , and M. Jay , “Obesity Management in Adults: A Review,” JAMA 330 (2023): 2000–2015. 38015216 10.1001/jama.2023.19897 PMC11325826

[2] T. D. Müller , M. Blüher , M. H. Tschöp , and R. D. DiMarchi , “Anti‐Obesity Drug Discovery: Advances and Challenges,” Nature Reviews Drug Discovery 21 (2022): 201–223. 34815532 10.1038/s41573-021-00337-8 PMC8609996

[3] M. Bossart , M. Wagner , R. Elvert , et al., “Effects on Weight Loss and Glycemic Control With SAR441255, a Potent Unimolecular Peptide GLP‐1/GIP/GCG Receptor Triagonist,” Cell Metabolism 34 (2022): 59–74.e10. 34932984 10.1016/j.cmet.2021.12.005

